# Comparative secretomics reveals novel virulence-associated factors of *Vibrio parahaemolyticus*

**DOI:** 10.3389/fmicb.2015.00707

**Published:** 2015-07-17

**Authors:** Yu He, Hua Wang, Lanming Chen

**Affiliations:** ^1^Key Laboratory of Quality and Safety Risk Assessment for Aquatic Products on Storage and Preservation (Shanghai), China Ministry of Agriculture, College of Food Science and Technology, Shanghai Ocean UniversityShanghai, China; ^2^Department of Food Science and Technology, The Ohio State UniversityColumbus, OH, USA

**Keywords:** *Vibrio parahaemolyticus*, virulence factor, secretome, aquatic products, vaccine candidate

## Abstract

*Vibrio parahaemolyticus* is a causative agent of serious human seafood-borne gastroenteritis disease and even death. In this study, for the first time, we obtained the secretomic profiles of seven *V. parahaemolyticus* strains of clinical and food origins. The strains exhibited various toxic genotypes and phenotypes of antimicrobial susceptibility and heavy metal resistance, five of which were isolated from aquatic products in Shanghai, China. Fourteen common extracellular proteins were identified from the distinct secretomic profiles using the two-dimensional gel electrophoresis (2-DE) and liquid chromatography tandem mass spectrometry (LC-MS/MS) techniques. Of these, half were involved in protein synthesis and sugar transport of *V. parahaemolyticus*. Strikingly, six identified proteins were virulence-associated factors involved in the pathogenicity of some other pathogenic bacteria, including the translation elongation factor EF-Tu, pyridoxine 5′-phosphate synthase, σ^54^ modulation protein, dihydrolipoyl dehydrogenase, transaldolase and phosphoglycerate kinase. In addition, comparative secretomics also revealed several extracellular proteins that have not been described in any bacteria, such as the ribosome-recycling factor, translation elongation factor EF-Ts, phosphocarrier protein HPr and maltose-binding protein MalE. The results in this study will facilitate the better understanding of the pathogenesis of *V. parahaemolyticus* and provide data in support of novel vaccine candidates against the leading seafood-borne pathogen worldwide.

## Introduction

*Vibrio parahaemolyticus* is a Gram-negative bacterium that thrives in marine, estuarine and aquaculture environments worldwide (Broberg et al., 2011; Letchumanan et al., 2014). The bacterium is a causative agent of serious human seafood-borne gastroenteritis disease and even death (Boyd et al., 2008; Ceccarelli et al., 2013). Previous research indicated that pathogenic bacteria establish infection, elicit diseases and survive in hostile environments via a large armamentarium of virulence mechanisms (Chen et al., 2012). Pathogenic *V. parahaemolyticus* strains have been reported to produce two major toxic proteins, thermostable direct haemolysin (TDH) and TDH-related haemolysin (TRH), in the human gastrointestinal tract where it elicits diarrhea disease (Boyd et al., 2008). Nevertheless, approximately 90–99% of *V. parahaemolyticus* isolates of environmental origins were detected negative for the two major toxic genes (e.g., Su and Liu, 2007; Martinez-Urtaza et al., 2008; Flores-Primo et al., 2014; Haley et al., 2014; Letchumanan et al., 2015). Environmental isolates lacking *tdh* and/or *trh* are also highly cytotoxic to human gastrointestinal cells, indicating other virulence factors exist (Raghunath, 2014). Due to the intricacy of the pathogenesis of *V. parahaemolyticus*, there exists a clear need for a complete understanding of the other virulence-associated factors of the bacterium.

Virulence factors are secreted by bacterial secretion systems (Wooldridge, 2009). Numerous previous studies have been conducted to characterize effector proteins secreted by toxic *V. parahaemolyticus* isolates, which possess virulence-associated type III and type VI secretion systems (T3SS and T6SS) (Makino et al., 2003). Recently, several T3SS-secreted proteins have been characterized, which manipulated host cell mitogen activated protein kinase for critical steps in the pathogenesis of *V. parahaemolyticus* (Ono et al., 2006; Matlawska-Wasowska et al., 2010). For example, VopQ encoded by the *VP1680* gene of *V. parahaemolyticus* RIMD2210633 (GenBank accession no. NC_004603.1, NC_004605.1) induced PI3-kinase-independent autophagy and antagonized phagocytosis (Burdette et al., 2009), while VopS (*VP1686*) induced toll-like receptor-independent apoptosis in macrophage through NF-kappaB inhibition (Bhattacharjee et al., 2006; Yarbrough et al., 2009). They also suppressed inflammasome activation by interfering with host autophagy signaling (Higa et al., 2013). In addition, VopV (*Vpa1357*) was an F-actin-binding effector and required for *V. parahaemolyticus*-induced enterotoxicity (Hiyoshi et al., 2011), while VopZ (*Vpa1336*) mediated pathogenesis by independently enabling intestinal colonization and inhibiting the transforming growth factor β-activated kinase activation (Zhou et al., 2013). Recent research also revealed cell density- and quorum sensing-dependent expression of T6SS2 in *V. parahaemolyticus* (Wang et al., 2013).

To date, very few information is available in secreted proteins of non-toxic bacterial strains, including *V. parahaemolyticus*. Herein, for the first time, we investigated the secretomes of seven *V. parahaemolyticus* strains of clinical and food origins. The strains exhibited various toxic genotypes and resistant phenotypes. Fourteen common extracellular proteins were characterized using the two-dimensional gel electrophoresis (2-DE) and liquid chromatography tandem mass spectrometry (LC-MS/MS) techniques, most of which have not been reported previously in *V. parahaemolyticus*. The information in this study sheds light on the pathogenesis of the leading seafood-borne pathogen worldwide.

## Materials and methods

### *V. parahaemolyticus* isolation and identification

*V. parahaemolyticus* isolation and identification were performed according to the methods described previously (Song et al., 2013). Aquatic products including shrimps and shellfish were sampled from fish markets in Shanghai, China in 2011–2014. DNA sequencing was carried out by Shanghai Sangon Biological Engineering Technology and Services Co., Ltd. (Shanghai, China). The 16S rDNA sequences of the strains identified in this study were deposited in the GenBank database under the accession numbers from KP696473 to KP696477.

### Susceptibility to antimicrobial agents and heavy metals

*V. parahaemolyticus* strains were measured for *in vitro* susceptibility to 10 antimicrobial agents and heavy metals according to the methods described previously (Song et al., 2013). The heavy metals used in this study included: NiCl_2_, CrCl_3_, CdCl_2_, PbCl_2_, CuCl_2_, ZnCl_2_, MnCl_2_ and HgCl_2_ (Sinopharm Chemical Reagent Co., Ltd, Shanghai, China).

### Pulsed-field gel electrophoresis (PFGE) analysis

*V. parahaemolyticus* strains were individually cultured in Luria-Bertani (LB) broth (Beijing Land Bridge Technology Co., Ltd., Beijing, China) (pH 7.0, 1% NaCl) aerobically at 37°C with shaking at 180 rpm overnight. Genomic DNA was isolated using the CHEF Bacterial DNA Plug Kit (Bio-Rad Laboratories Inc., Hercules, CA, USA) according to the manufacturer's instructions. Briefly, each agarose plug was prepared by mixing an equal volume of the bacterial cell culture and melted 2% CleanCut agarose provided by the Kit, and then digested with *Not*I restriction endonulease (Japan TaKaRa BIO, Dalian Company, Dalian, China) at 37°C overnight according to the manufacturer's instructions. The resulting DNA fragments were resolved using the CHEF Mapper system (Bio-Rad Laboratories Inc.) with 0.5 × Tris-borate ethylene diamine tetraacetic acid (EDTA) buffer (Shanghai Sangon Biological Engineering Technology and Services Co., Ltd.). Electrophoresis was performed at 6 V/cm at 14°C with a field angle of 120° using 1% SeaKem® Gold Agarose gel (Lonza, Basel, Switzerland). The pulse time was ramped from 10 to 35.03 s over 19 h. Following electrophoresis, the gels were stained with 0.5 μg/ml ethidium bromide (Shanghai Sangon Biological Engineering Technology and Services Co., Ltd.). The PFGE patterns were visualized under short-wavelength UV light (260 nm) and imaged using the UVP EC3 Imaging system (UVP LLC, Upland, CA, USA).

### Isolation of extracellular proteins of *V. parahaemolyticus* strains

Extracellular proteins of *V. parahaemolyticus* strains were isolated according to the method described by Ono et al. with minor modifications (Ono et al., 2006). Briefly, *V. parahaemolyticus* strains were individually cultured in the LB broth (pH 8.5, 3% NaCl) to mid-logarithmic phase at 37°C without shaking. Growth curves were determined using the Synergy 2 Multi-Mode Microplate Reader (BioTek Instruments, Inc., Winooski, VT, USA) as described previously (Sun et al., 2014). Bacterial protease inhibitors complex (Shanghai Sangon Biological Engineering Technology and Services Co., Ltd.) was added to each bacterial cell culture, which was then centrifuged at 12,000 rpm for 5 min at 4°C. The supernatant was filtered through 0.22 μm pore-size membranes (Millipore, Bedford, MA, USA) to remove residual bacterial cells. The filtrate was precipitated by adding trichloracetic acid (TCA) to a final concentration of 10% (vol/vol), and then incubated on ice overnight. The extracellular proteins were collected by centrifugation at 12,000 rpm for 30 min at 4°C. The pellet was washed with ice-cold acetone for five times, air-dried and stored at −80°C. Protein concentration was measured using the BCA Protein Assay Kit (Shanghai Sangon Biological Engineering Technology and Services Co., Ltd.) with bovine serum albumin as the standard.

### 2-DE analysis

The 2-DE analysis was performed according to the manufacturer's instructions for *E. coli* (Bio-Rad Laboratories Inc.) with slight modifications such as bacterial culture and collection conditions. Extracellular protein pellets were individually dissolved with 1 ml of rehydration solution containing 8 M urea, 4% (wt/vol) 3-[3-cholamidopropyl-dimethylammonio-1-propanesulfonate (CHAPS) (Bio-Rad Laboratories Inc.)], 2 mM tributyl phosphine (TBP) (Bio-Rad Laboratories Inc.), 0.2% (vol/vol) Bio-Lyte 3/10 ampholyte (Bio-Rad Laboratories Inc.) and 0.0002% (wt/vol) bromophenol blue, and then centrifuged at 12,000 rpm for 10 min at room temperature to remove undissolved residues. Isoelectric focusing (IEF) was performed as the first-dimension separation with the immobilized pH gradient gel (IPG) strip (pH 3-10/NL, 17 cm) (Bio-Rad Laboratories Inc.). Aliquots of each 300 μl of protein sample were individually applied to the strips (pH 3-10/NL, 17 cm) and positive rehydrated for 17 h at 17°C. After rehydration, IEF of the samples was run with a 8-step program: 50, 100, 500 and 1000 V for 1 h with slow ramping, 2000 and 4000 V for 1 h with linear ramping, 6000 V for 3 h with rapid ramping, and 10,000 V with rapid ramping until 70,000 V-hr was reached. Following the electrophoresis in the first dimension, the IPG gel strip was incubated in an equilibration buffer I [6 M urea, 0.05 M Tris, 2% SDS, 20% glycerol, 2% (wt/vol) dithiothreitol] for 15 min, and then washed for a further 15 min with an equilibration buffer II [6 M urea, 0.05 M Tris, 2% SDS, 20% glycerol, 2.5% (wt/vol) iodoacetamide (Sigma-Aldrich, MO, USA)].

The second-dimension separation was performed by sodium dodecyl sulfate-polyacrylamide gel electrophoresis (SDS-PAGE) with 15% separation gel using a Mini-PROTEANW electrophoresis cell (Bio-Rad Laboratories Inc.) as described previously (Shi et al., 2013). After electrophoresis, the gel was stained using the Silver Stain Plus Kit (Bio-Rad Laboratories Inc.) according to the manufacturer's instructions. The stained gels were stored in 1% acetic acid at 4°C. The assays were performed in triplicates. The PDQuest Advanced-8.0.1 software (Bio-Rad Laboratories Inc.) was used for the detection and analysis of protein spots on the 2-DE profiles.

### LC-MS/MS analysis

The visible and discriminative protein spots were individually excised from the 2-DE gels and then digested by freshly prepared Sequencing Grade Modified Trypsin (Promega, Madison, WI, USA) at 37°C overnight. The resulting peptides were analyzed at Beijing Protein Innovation (Beijing, China) using liquid chromatography tandem mass spectrometry (LC-MS) with a serially coupled microcolumn in a Paradigm HPLC system (Shimadzu Inc., Kyoto, Japan) coupled with a microTOF-QII mass spectrometer (Bruker, Billerica, Germany) (LC-MS/MS). The data collection was performed using the Bruker Data Analysis 4.0 software (Bruker, Billerica, MA, Germany).

### Data analysis

Data analysis was carried out at Beijing Protein Innovation. The collected LC-MS/MS data files were converted to the Mascot generic format (mgf). The combined mgf-files derived from triplicate analyses were sent to the Mascot server (Version 2.3.01, Matrix Science, London, UK) for automated peptide identification using the UniProt database. The following Mascot settings were used: carbamidomethyl was specified as a fixed modification; methionine oxidation was specified with variable modifications; two missed cleavages were allowed. The precursor and fragment mass tolerance was set to 0.1 Da, and the significance threshold was *p* < 0.05.

## Results

### *V. parahaemolyticus* isolation and identification

Aquatic products were sampled from fish markets in Shanghai in 2011–2014. Pure cultures of *V. parahaemolyticus* isolates were identified according to the instructions of the China Government Standard (GB17378-2007) and the Standard of the Bacteriological Analytical Manual (BAM) of U.S. Food and Drug Administration (8th Edition, Revision A, 1998). A total of 334 *V. parahaemolyticus* strains were obtained and detected positive for the thermolabile hemolysin gene (*tlh*) by polymerase chain reaction (PCR). Toxin-related genes were also examined by PCR. In most cases, the *V. parahaemolyticus* strains were featured as not virulent, since amplification of toxic *tdh* gene was negative, whereas approximately 97.1% of the isolates yielded no product for the *trh* gene. Secretomes of five representative *V. parahaemolyticus* strains isolated from the most common shrimps sold in Shanghai, China were assessed in this study (Table 1). Of these, *V. parahaemolyticus* Chn201 and Chn204 strains were detected as the *trh*-positive genotype, while the other strains were negative for the toxic genes (Table 1).

**Table 1:** **Toxic genotypes and phenotypes of antimicrobial susceptibility and heavy metal resistance of the** ***V. parahaemolyticus*** **strains**.

**Strains**	**Source**	**Year of islation**	***tlh***	***tdh***	***trh***	Resistance to antibiotics^*^	Resistance to heavy metals^**^
*V. parahaemolyticus* Chn201	*Moerella iridescens*, China	2014	+	−^***^	+	−	−
*V. parahaemolyticus* Chn204	*Haliotis asinina*, China	2014	+	−	+	AMP	Zn
*V. parahaemolyticus* Chn214	*Metapenaeus ensis*, China	2013	+	−	−	AMP, STR	Cd, Cu
*V. parahaemolyticus* Chn289	*White prown*, China	2013	+	−	−	AMP	Hg
*V. parahaemolyticus* Chn25	Shrimps, China	2011 (Song et al., 2013)	+	−	−	SXT, STR	−
*V. parahaemolyticus* ATCC33847	Gastroenteritis, Maryland, USA	1973 (Baumann et al., 1973)	+	+	−	AMP	−
*V. parahaemolyticus* ATCC17802	Shirasu food poisoning, Japan	1965 (Fujino et al., 1965)	+	−	+	−	−

* *AMP, ampicillin; STR, streptomycin; SXT, sulfamethoxazole-trimethoprim*.

** *Zn, zinc; Cd, cadmium; Cu, copper; Hg, mercury*.

*** *-, not detected*.

### PFGE-based genotypes of the *V. parahaemolyticus* strains

**Figure 1:**
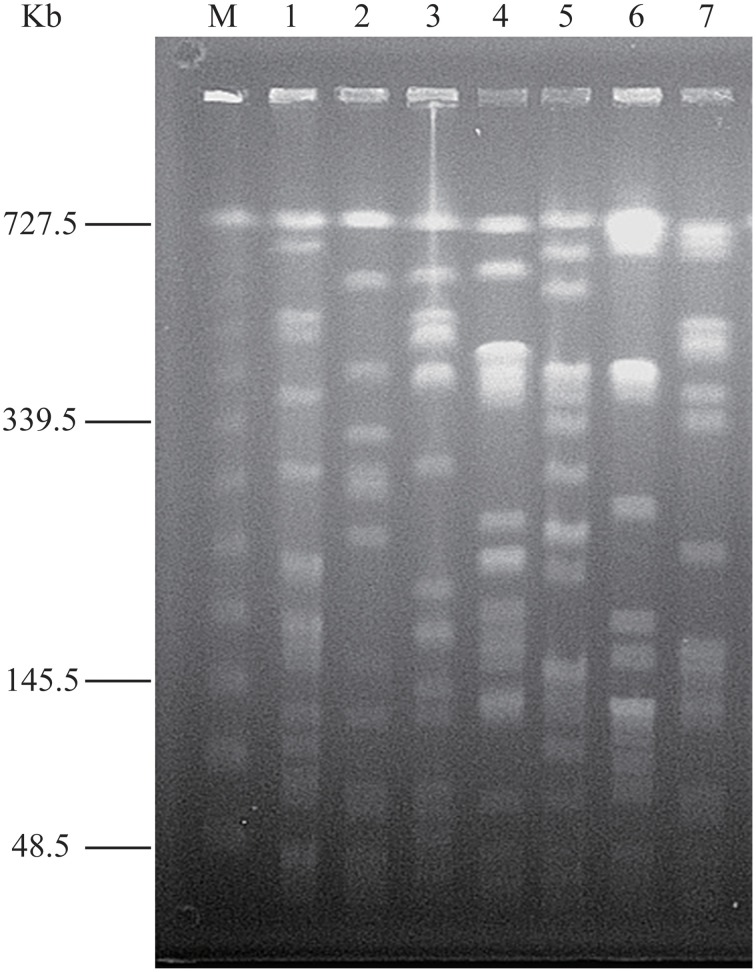
***Not*****I-PFGE genotyping of the** ***V. parahaemolyticus*** **strains**. Lane M: Lambda Ladder PFG Marker (48.5–727.5 kb, New England Biolabs, Beijing Company, Beijing, China); Lane 1-7: *V. parahaemolyticus* Chn25, ATCC17802, Chn201, Chn204, Chn214, Chn289 and ATCC33847 strains, respectively.

In order to compare the secretomes of toxic and non-toxic *V. parahaemolyticus* strains, we used two standard toxic strains in this study, including the ATCC33847 (*tdh*^+^*trh*^−^) and ATCC17802 (*tdh*^−^*trh*^+^) isolated from clinical and food-poisoning cases, respectively (Fujino et al., 1965; Baumann et al., 1973). Genomic DNA of the seven *V. parahaemolyticus* strains was individually digested with the restriction endonuclease *Not*I, and the resulting DNA fragments were resolved by PFGE. This analysis revealed different genomic fingerprints of the strains tested (Figure 1). Clustering analysis of the genomic fingerprints revealed seven distinguishable *Not*I-PFGE types, demonstrating that the strains chosen for the secretomic analysis exhibited various genotypes.

### Antimicrobial susceptibility and heavy metal resistance of the *V. parahaemolyticus* strains

Antimicrobial susceptibility of the *V. parahaemolyticus* strains were determined and the results were summarized in Table 1. All the strains were susceptible to three of the 10 antimicrobial agents tested, including chloramphenicol, gentamicin and tetracycline. Of these, Chn201 (*tdh*^−^*trh*^+^) and ATCC17802 (*tdh*^−^*trh*^+^) strains were susceptible to all the 10 drugs. In contrast, ampicillin resistance was the most predominant among the strains examined in this study.

Susceptibility of the strains to heavy metals including cadmium (Cd), chromium (Cr), copper (Cu), mercury (Hg), manganese (Mn), nickel (Ni), lead (Pb) and zinc (Zn) was also determined (Table 1). Chn214 strain displayed resistance phenotypes to Cd and Cu, while Chn204 and Chn289 strains resistance to Zn and Hg, respectively. In contrast, all the strains were susceptible to the four heavy metals tested, including Ni, Cr, Mn and Pb. Moreover, Chn25, Chn201, ATCC33847 and ATCC17802 strains showed no resistance to all the heavy metals tested.

### Secretome profiles of the *V. parahaemolyticus* strains

To gain an insight into the possible growth dynamics of the *V. parahaemolyticus* strains with various genotypes and phenotypes, we determined growth curves of the bacteria cultured in LB broth (pH 8.5, 3% NaCl) at 37°C without shaking. As illustrated in Figure S1 (Supplementary Material), the growth of ATCC33847 stain of clinical origin was notably slower than the other strains. However, no obvious difference in growth was observed under the same condition among the other strains.

**Figure 2:**
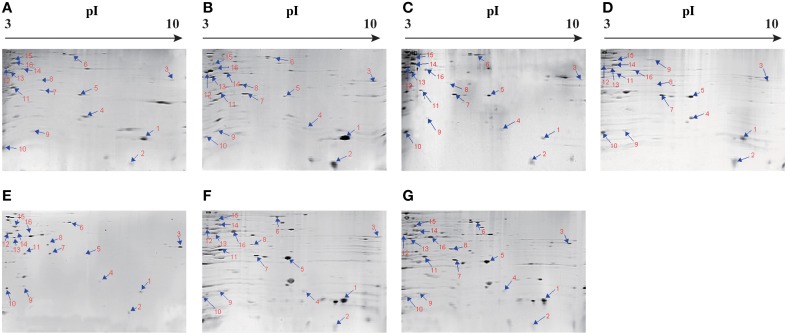
**Isolation of extracellular proteins of the** ***V. parahaemolyticus*** **strains by 2-DE. (A–G):** *V. parahaemolyticus* Chn201, Chn204, Chn214, Chn289, Chn25, ATCC33847 and ATCC17802, respectively. The protein spots marked with the same numbers in red were characterized by LC-MS/MS analysis. pI, isoelectric point.

The supernatant of the cultures at mid-logarithmic growth phase with OD_600nm_ values ranging from 0.5 to 0.6 were collected, and extracellular proteins were isolated, and analyzed by 2-DE. This analysis uncovered distinct secretome profiles (Figure 2), showing various numbers of visible protein spots. The patterns yielded from three independent 2-DE gels per biological sample were consistent (the data not shown). Based on the consensus patterns, one interesting observation was that toxic ATCC33847 and ATCC17802 strains appeared to secret more extracellular proteins (56–59) than the five strains derived from aquatic products (20–36), including Chn201 and Chn204 strains with the *trh*^+^ toxic genotype. In addition, 16 protein spots, designated vps1 to vps16, were observed at similar locations on all the 2-DE patterns derived from the strains tested (Figure 2, marked with the same numbers). These protein spots were excised from the 2-DE gels and digested with the trypsin. The resulting peptides were further identified by LC-MS/MS.

### Identification of common extracellular proteins of the *V. parahaemolyticus* strains

The results yielded from LC-MS/MS analysis were summarized in Table 2. Total 14 protein sequences were successfully obtained. The metabolism-related proteins constituted the largest proportion of the identified proteins shared among the *V. parahaemolyticus* strains tested. Of these, strikingly, approximately 43% were associated with protein synthesis of the bacterium. For example, the protein spots vps3 and vps4 were identified as the components S2 and S6 of the 30S subunit of bacterial ribosome, respectively, while the vps9 was the component L9 of the 50S subunit. In addition, the vps5 was a ribosome-recycling factor (RRF), moreover, two other translation-associated proteins, vps15 and vps16, were also found. The former was an elongation factor Tu (EF-Tu), while the latter matched an elongation factor Ts (EF-Ts), a guanosine nucleotide exchange factor for EF-Tu.

**Table 2:** **Identification of the protein spots from vps1 to vps16 on the secretome profiles by LC-MS/MS analysis**.

**Protein spot No**.	**Uniprot No**.	**Protein**	**Gene**	MW (Da)^*^	**pI**	**Score**	**Sequence coverage**	**Putative function**
vps1	Q87S68	Sigma-54 modulation protein	*VP0556*	12,435	6.85	524	48%	Primary metabolic process
vps2	Q87RJ9	Phosphocarrier protein HPr	*VP0795*	9103	6.25	89	42%	Carbohydrate transmembrane transport, cytoplasm
vps3	W6DNZ4	30S ribosomal protein S2	*rpsB*	26,791	8.59	668	47%	Structural constituent of ribosome, translation, intracellular
vps4	D0M6F3	30S ribosomal protein S6	*rpsF*	14,974	6.23	350	50%	rRNA binding, structural constituent of ribosome, translation
vps5	W6DD11	Ribosome-recycling factor	*frr*	20,558	6.07	767	12%	Translational termination, translation, cytoplasm
vps6	W6DHC2	Dihydrolipoyl dehydrogenase	*VPUCM_2603*	51,299	5.65	61	14%	Glycolytic process, cell redox homeostasis, detoxification of mercury ion, cytoplasm, oxidoreductase activity
vps7	Q87S68	Sigma-54 modulation protein	*VP0556*	12,435	6.85	92	20%	Primary metabolic process
vps8	W6DKZ7	Pyridoxine 5′-phosphate synthase	*pdxJ*	26783	5.33	165	19%	Catalytic activity, transferase activity, transferring nitrogenous groups, cytoplasm
vps9	W6DLE7	50S ribosomal protein L9	*rplI*	15699	5.19	2016	32%	rRNA binding, structural constituent of ribosome, translation, intracellular
vps10	Q87PP7	Hypothetical protein	*VP1454*	16908	5.15	64	9%	Cell outer membrane, integral component of membrane
vps11	W6DWX7	Transaldolase	*tal*	35012	4.86	69	6%	Catalytic activity, transferase activity, cytoplasm
vps12	Q87GB6	Maltose ABC transporter	*Vpa1401*	42114	4.84	1489	51%	Transporter activity, maltose transmembrane transporter activity
vps13	W6DWX7	Transaldolase	*tal*	35012	4.86	999	56%	Catalytic activity, transferase activity, cytoplasm
vps14	W6DHT7	Phosphoglycerate kinase	*pgk*	41049	4.9	1212	50%	Nucleotide binding, kinase activity, phosphoglycerate kinase activity, ATP binding, transferase activity, cytoplasm
vps15	S5JFA8	Elongation factor Tu	*tuf*	43321	4.8	2732	42%	Translational elongation factor activity, nucleotide binding, GTPase activity, GTP binding, intracellular, cytoplasm
vps16	S5IN04	Elongation factor Ts	*tsf*	29869	5.18	1738	56%	Translation elongation factor activity, intracellular, cytoplasm

* *Molecular weight*.

The protein spot vps8 was identified as a pyridoxine 5′-phosphate synthase (PdxJ) that catalyzes the final-step reaction of vitamin B_6_ *de novo* synthesis, a complicated ring closure reaction between the two acyclic compounds 1-deoxy-D-xylulose-5-phosphate and aminoacetone-3-phosphate to form pyridoxine 5′-phosphate and inorganic phosphate (Franco et al., 2001).

Among the identified extracellular proteins, two were involved in carbohydrate transport across cellular membranes in bacteria. The protein spot vps2 matched a phosphocarrier protein HPr, an essential component of the multicomponent phosphotransferase system, by which many sugars are transported into bacteria across the cytoplasmic membrane, concomitantly phosphorylated and then fed into glycolysis (Postma et al., 1993). In addition, the vps12 was a periplasmic maltose-binding protein MalE of the maltose ATP binding cassette (ABC) transporter (Davidson and Maloney, 2007), which also contains integral cytoplasmic membrane proteins MalF and MalG, and two copies of the ATPase subunit MalK (Boos and Lucht, 1996). To our knowledge, none of the secreted HPr and MalE derived from any toxic and non-toxic bacteria has been described thus far. In this study, our data implied possible unknown regulation mechanisms underlying the key sugar transports in central carbohydrate metabolism, which was mediated by the membrane-linked HPr and MalE in *V. parahaemolyticus*.

Several identified extracellular proteins have been reported to be involved in the virulence of some pathogenic bacteria. For example, in this study, two protein spots (vps1 and vps7) were identified as a Sigma-54 (σ^54^) modulation protein of *V. parahaemolyticus*, while the vps6 was a dihydrolipoyl dehydrogenase (DLDH). In addition, transaldolase (vps11 and vps13) can be recruited on the cell surface, and secreted via a non-classical secretion mechanism or an uncharacterized translocation pathway (González-Rodríguez et al., 2012). Moreover, the vps14 was found as a phosphoglycerate kinase (PGK) that catalyzes the transfer of a phosphate group from 1,3-diphosphoglycerate to ADP to produce 3-phosphoglycerate and ATP (Smith et al., 2011). Taken together, in this study, our data suggested that the extracellular proteins of σ^54^, DLDH, transaldolase and PGK perhaps also contributed to the pathogenecity of *V. parahaemolyticus*. Further studies on these potential virulence-associated factors will improve our understanding of the pathogenesis of the bacterium.

In addition, the protein spot vps10 matched a hypothetical protein (9% sequence coverage, 64 score) encoded by the *VP1454* gene of *V*. *parahaemolyticus* RIMD2210633 with currently unknown functions in the public databases.

## Discussion

In this study, approximately 2.9% of the *V. parahaemolyticus* strains isolated from aquatic products sampled from fish markets in Shanghai in 2011–2014 carried the toxic *trh* gene, but none had the toxic *tdh* gene, consistent with previous reports (Su and Liu, 2007; Martinez-Urtaza et al., 2008; Flores-Primo et al., 2014). Antimicrobial susceptibility and heavy metal resistance of the *V. parahaemolyticus* strains chosen for the comparative secretome analysis also correlated with those reported by Song et al. (2013), where ampicillin resistance was the most predominant amongst the *Vibrios* isolated from Shanghai fish markets in 2011. Moreover, compared with the prior work (Song et al., 2013), the data in this study revealed similar but narrow heavy metal resistance patterns, although based on a fairly small number of isolates analyzed here.

Distinct secretomic profiles derived from the seven *V. parahaemolyticus* strains of clinical and food origins as well as the LC-MS/MS analysis revealed approximately 43% of the common extracellular proteins involved in protein synthesis of the bacterium. Bacterial ribosome is a large protein-RNA complex that consists of two major subunits (a small 30S and a large 50S subunits), each of which is composed of a variety of proteins (Brodersen et al., 2002). The components S2 (vps3), S6 (vps4) and L9 (vps9) are associated with the formation of the translation initiation complex and possibly interact with mRNA and other components of the ribosome (Lieberman et al., 2000; Brodersen et al., 2002; Wei et al., 2014). The S2 and ribosomal protein L7/L12 were also detected as extracellular proteins of *Haemophilus parasuis* and *Streptococcus suis* by 2-DE analysis (Wu et al., 2008b; Wei et al., 2014). Despite highly conserved translational machinery with invariable rRNA and protein components, the formation of distinct ribosomal subpopulations has been reported in bacteria when encountered adverse conditions (Moll and Engelberg-Kulka, 2012). It will be interesting to investigate the biological function of the ribosomal proteins of *V. parahaemolyticus* in the future research. In addition, the RRF (vps5) is responsible for the dissociation of ribosomes from messenger RNA after the termination of translation (Li et al., 2010). It has been characterized as an essential protein for the survival of *Escherichia coli* (Selvaraj et al., 2013). In this study, two other translation-associated proteins, vps15 (EF-Tu) and vps 16 (EF-Ts), were also identified. These two proteins are important components of the multistep ribosomal decoding pathway that ensures rapid and accurate translation (Burnett et al., 2013; Yikilmaz et al., 2014). It has been reported that EF-Tu of *Streptococcus pneumonia* was a new virulence factor that binds human complement factors, aids in immune evasion and host tissue invasion (Sarkar et al., 2013). To our knowledge, no linking to secreted proteins of the RRF and EF-Ts has been described previously.

The PdxJ (vps8) is absent from humans, but exists in many human pathogens, such as *E. coli*, *Cercospora* spp. and *Aspergillus nidulans*. The enzyme has been suggested as a potential target for the development of novel drugs to control the pathogens (Garrido-Franco, 2003).

Previous studies have revealed that the σ^54^ (vps1 and vps7) played an important role in the virulence of *Pseudomonas syringae*, *Erwinia carotovora* and *Pseudomonas aeruginosa* (Hendrickson et al., 2000, 2001; Chatterjee et al., 2002). It was also essential for biofilm formation in *Vibrio fischeri* (Wolfe et al., 2004), and bacterial quorum sensing regulation and pilin and flagellin production in *Vibrio cholerae* (Prouty et al., 2001; Ishikawa et al., 2009). In this study, the protein vps6 was identified as a DLDH. It was also detected as an extracellular enzyme secreted by *Bacillus subtilis* WY34 (Wu et al., 2008a), and identified as a virulence-associated determinant of *Streptococcus pneumoniae* and *Mycoplasma gallisepticum* (Smith et al., 2002; Hudson et al., 2006). DLDH could be recognized by anti-sera as one of the cell wall-associated proteins isolated from *Staphylococcus epidermidis* (Sellman et al., 2005). Recently, western blot analyses of whole cell lysates of *Vibrio harveyi* and *Vibrio alginolyticus* suggested that DLDH was a novel immunogenic protein (Pang et al., 2010, 2013). In addition, the extracellular transaldolase [vps11 (6% sequence coverage, 69 score) and vps13] of *Bifidobacterium bifidum* A8 was demonstrated *in vitro* as a specific aggregation factor contributing to the strong auto-aggregation phenotype of the bacterium. When exposed on the cell surface, it could act as an important colonization factor for the survival of *B. bifidum* in host intestinal tract (González-Rodríguez et al., 2012). In this study, the protein vps14 was identified as a PGK. In *Streptococcus agalactiae*, PGK is a major outer surface protein that can be recognized by host immune system, and the recombinant PGK has been used as an antigen in a neonatal-animal model against *S. agalactiae* infection (Hughes, 2002; Wang et al., 2014). PGK has also been suggested as a potential vaccine candidate against *Lactococcus garvieae* infection and *Streptococcus suis* biofilm formation (Shin et al., 2007; Valette et al., 2012).

Overall, this study constitutes the first investigation of the secretomes of seven *V. parahaemolyticus* strains with toxic and non-toxic genotypes. Fourteen common extracellular proteins were identified from the distinct secretomic profiles derived from the strains by 2-DE and LC-MS/MS analysis. Of these, half were involved in protein synthesis and sugar transport of *V. parahaemolyticus*. Notably, six identified proteins were virulence-associated factors involved in the pathogenicity of some other pathogenic bacteria, including the EF-Tu, PdxJ, σ^54^, DLDH, transaldolase and PGK. In addition, comparative secretomics also uncovered several extracellular proteins that have not been described in any bacteria, such as the ribosome-recycling factor, EF-Ts, HPr and MalE. Our secretome data, coupled with the previous studies, indicated that bacterial virulence-associated factors fall into three distinct categories. One of these exhibits strong strain-specificity and plays a crucial role in the virulence of the pathogens, e.g., the TDH and TRH in *V. parahaemolyticus*, as well as the cholera toxin (CTX) in *V. cholerae*. The EF-Tu, PdxJ, σ^54^, DLDH, transaldolase and PGK fall into the second category, the members of which are likely essential for the colonization, invasion and survival of the pathogens in host environments, such as human gastrointestinal and respiratory tracts. The third category may contain the secreted proteins involved in the key cellular synthesis and metabolism, e.g., EF-Ts, HPr and MalE in *V. parahaemolyticus*. Given that the members belonging to the latter two categories were also identified from the non-toxic *V. parahaemolyticus* strains, in the future research, it will be interesting to further address their functions in the virulence of the bacterium, particularly in the complex gastrointestinal flora. The results in this study will facilitate the better elucidation of the pathogenecity of *V. parahaemolyticus*, and also provide data in support of the development of novel vaccine against the leading seafood-borne pathogen worldwide.

## Author contributions

YH, HW, LC participated in the design and or discussion of the study. YH carried out the major experiments. YH, LC analyzed the data. LC wrote the article. HW revised it for important intellectual improvement. All authors read and approved the final version to be published.

### Conflict of interest statement

The authors declare that the research was conducted in the absence of any commercial or financial relationships that could be construed as a potential conflict of interest.

## Supplementary material

Figure S1 **Growth curves of the** ***V. parahaemolyticus*** **strains**. The strains were cultured in LB broth (pH 8.5, 3% NaCl) at 37°C without shaking, and growth curves were determined using Synergy 2 Multi-Mode Microplate Reader.
